# Blood biomarkers for Alzheimer's disease are correlated with treatment outcomes in a randomized trial assessing the effects of escitalopram on agitation

**DOI:** 10.1002/alz70856_104004

**Published:** 2025-12-26

**Authors:** Maansi Barnwal, Sheriza Baksh, David Shade, Abhay Moghekar, Paul B. Rosenberg, Anton P. Porsteinsson, Kostas G. Lyketsos

**Affiliations:** ^1^ Johns Hopkins University School of Medicine, Baltimore, MD, USA; ^2^ Johns Hopkins University Bloomberg School of Public Health, Baltimore, MD, USA; ^3^ University of Rochester School of Medicine and Dentistry, Rochester, NY, USA; ^4^ School of Medicine, Johns Hopkins University, and Johns Hopkins Bayview Medical Center, Baltimore, MD, USA

## Abstract

**Background:**

Escitalopram for Agitation in Alzheimer's Disease (S‐CitAD) was an NIH‐funded RCT that randomized 173 participants with Alzheimer's Disease (AD) and agitation to escitalopram or placebo for 12 weeks assessing efficacy for clinically significant agitation. There was no significant advantage for escitalopram in treating agitation. This might be attributed to including participants at various stages of AD brain pathology that would be reflected in levels of blood biomarkers.

**Methods:**

We examined associations of baseline blood biomarkers and clinical measures at baseline and follow up regarding: (1) agitation severity (NPI‐C‐A+A); (2) cognitive status, (MMSE); and (3) escitalopram treatment. Abeta42, Abeta40, GFAP, NfL were measured using SIMOA N4PE assays, and pTau217 using the Alzpath‐pTau217 SIMOA on Quanterix HD‐X.

**Results:**

Abeta40, Abeta42, pTau217, GFAP, and NfL were available for 82 participants. pTau217>0.42 pg/ml and Abeta42/40<0.04 pg/ml indicated significant amyloid pathology. Of 82 participants with pTau217 data, 77 (94%) scored above threshold supporting the clinical diagnosis of AD. In contrast, only 47 were below threshold for Abeta42/40. There was 61% agreement between biomarker cut‐offs for pTau217 and Abeta42/40.

No baseline associations between biomarkers and NPI‐C‐A+A scores were significant (*p* >0.05). Baseline higher pTau217 predicted higher NPI‐C‐A+A scores at week 6 (beta=3.26, *p* <0.001) and week 12 (beta=2.86, *p* = 0.01) after randomization.

Baseline associations between Abeta40, Abeta42, NfL and MMSE scores were not significant (*p* >0.05). However, baseline higher levels of GFAP (beta=‐0.02, *p* = 0.0002) and pTau217 (beta=‐2.68, *p* = 0.003) were associated with lower baseline MMSE scores.

Baseline Abeta40, GFAP and NfL were not associated with treatment outcomes. After adjusting for treatment, higher baseline pTau217 was associated with greater odds of worsening NPI‐C‐A+A scores at weeks 6 (OR=2.79, *p* = 0.02) and 12 (OR=2.55, *p* = 0.02) compared to baseline.

**Conclusion:**

Baseline blood levels of pTau217 confirmed the presence of significant AD brain amyloid pathology in 94% of participants. Independent of treatment assignment, higher baseline blood levels of pTau217 predicted lower baseline MMSE scores and higher agitation severity at weeks 6 and 12 after randomization.

